# Nervous Athonia Cured by Electricity

**Published:** 1844-12-28

**Authors:** 


### NERVOUS APHONIA CURED BY ELECTRICITY.

BY M. PELLEGRINI.

A healthy man, 23 years of age, was imprisoned for having killed a friend in a quarrel. Three days afterwards he had a severe fit of epilepsy, and lost his voice. Active treatment had no effect on the aphonia, though it removed the epileptic tendency. The larynx seemed to have lost the power of motion; the tongue was moved with difficulty, was somewhat swollen, and drier than natural. Sixteen months after the attack electricity was employed. A voltaic pile of fifty plates were used, the zinc wire of which was applied to the region of the first vertebra, the copper wire to the tongue and glottis. One hundred shocks were passed through these parts the first day, and two hundred the second. On its repetition an epileptic fit and syncope occurred, for which he was bled. When he recovered he was able to speak indistinctly in a hoarse voice. After eight sittings, during which the same number of shocks were given each time, the voice was completely restored, and the cure proved radical and complete.—*Ibid, from Jour. de Pharmacie.*

